# Do topical applications of bisphosphonates improve bone formation in oral implantology? A systematic review

**DOI:** 10.4317/medoral.21887

**Published:** 2017-06-18

**Authors:** Naroa Lozano-Carrascal, Oscar Salomó-Coll, Federico Hernández-Alfaro, Sergio-Alexandre Gehrke, Jordi Gargallo-Albiol, José-Luis Calvo-Guirado

**Affiliations:** 1 Clinical Instructor of the International Master in Oral Surgery (IMOS). International University of Catalonia (UIC), Barcelona, Spain; 2 Assistant Professor of the International Master in Oral Surgery (IMOS). International University of Catalonia (UIC), Barcelona, Spain; 3 Professor and Chairman of the Department of Oral and Maxillofacial Surgery. International University of Catalonia (UIC), Barcelona, Spain; 4 Professor and Director of Biotechnology Cathedra of the University Catholica San Antonio de Murcia (UCAM) Murcia, Spain; 5 Director and Associate Professor of the International Master in Oral Surgery (IMOS). International University of Catalonia (UIC), Barcelona, Spain; 6 Full Professor and Director of International Dentistry Research Cathedra. Faculty of Medicine & Dentistry, San Antonio Catholic University of Murcia (UCAM), Murcia, Spain

## Abstract

**Background:**

The aim of this systematic literature review was to evaluate the feasibility of topical bisphosphonate application for preserving/enhancing alveolar bone in oral implantology.

**Material and Methods:**

An electronic search was conducted in the PubMed/Medline, EMBASE, Scopus, Web of knowledge, and Google-Scholar databases for articles dated from January 2000 to December 2016. Two reviewers assessed the quality of the studies independently.

**Results:**

A total of 154 abstracts were identified, of which 18 potentially relevant articles were selected; a final total of nine papers were included for analysis. Comparison of the findings of the selected studies was made difficult by the heterogeneity of the articles, all of them animal research papers that showed heterogeneity in the methodologies used and a high or moderate risk of bias.

**Conclusions:**

The topical application of bisphosphonate solution would appear to favor new bone formation in alveolar defects, and boosts the regenerative capacities of biomaterials resulting in increased bone density.

** Key words:**Alveolar bone, bone regeneration, topical application, biomaterial, bisphosphonates.

## Introduction

Bisphosphonates are a group of drugs commonly used for the treatment of various bone diseases, including osteoporosis, malignant hypercalcemia, multiple myeloma, or Paget’s disease (1,2). Two groups of bisphosphonates are available, with different mechanisms of action: amino and non-amino-bisphosphonates. Non-amino-bisphosphonates, such as clodronate and etidronate, inhibit bone resorption primarily by inducing osteoclast apoptosis through the formation of intracellular metabolites in osteoclasts. Amino-bisphosphonates, such as pamidronate, alendronate or zoledronate, offer greater potency through the addition of a primary amino-nitrogenated base (-NH2) (3,4). These act by inhibiting farnesyl diphosphate (FPP) synthase, a key enzyme in the mevalonate pathway (5).

As a consequence of their high affinity for Ca2+ ions, bisphosphonates are rapidly cleared from circulation and target hydroxya-patite bone mineral surfaces *in vivo* at sites of active bone remodeling. Several experimental studies have demonstrated that these drugs reduce bone resorption by inhibiting the activity of mature osteoclasts and promoting their apotosis (6,7). They also inhibit the formation and recruitment of new osteoclasts, suppressing the osteoclasts’ multinucleated cells during the osteoclast differentiation process (8-11). In addition, recent experimental studies have demonstrated that some bisphosphonates enhance osteoblast differentiation and activity. For example, alendronate and clodronate seem to act directly on these cells, stimulating differentiation, proliferation, and bone formation/mineralization (12-15).

Traditionally, bisphosphonates have been administrated both intravenously and orally. In a Beagle dog study, Reddy *et al.* 1995 (16) observed that the systemic administration of bisphosphonates prevented the alveolar bone destruction associated with peri-odontal disease. However, in recent years a worrying correlation has emerged between osteonecrosis of the jaw (ONJ) and the systemic administration of bisphosphonates (17-20). Because of these potential risks of intravenous bisphosphonate administration, other methods have been proposed. Yaffe *et al.* (21-23) demonstrated that the topical application of bisphosphonates minimizes bone resorption following muco-periostial flap surgery. Shibutani *et al.* (24) observed that topical bisphosphonates inhibited the progression of alveolar bone resorption in peri-implantitis.

The aim of this systemic literature review was to evaluate the potential capacity of the topical application of bisphosphonates to preserve/enhance alveolar bone in oral implantology.

## Material And Methods

- Focused Question 

Based on the Preferred Reporting Items for Systematic Reviews and Meta-Analyses (PRISMA) guidelines, a specific answerable question was formulated according to Participants, Interventions, Control, Outcomes (PICO) recommendations: “Does the topical application of bisphosphonate solution improve bone preservation/regeneration in alveolar bone?” 

The PICO framework was as follows:

(P) Participants: samples that underwent treatment with topical applications of bisphosphonate solution.

(I) Type of intervention: the intervention of interest was the effect of the topical application of bisphosphonates on bone regeneration/preservation in alveolar defects.

(C) Control intervention: bone regeneration/preservation without topical application of bisphosphonates.

(O) Outcome measures: bone resorption, new bone formation and/or bone volume/tissue volume, radiographic/histologic changes with and without topical application of bisphosphonates.

A preliminary search for previous systematic reviews and meta-analyses was conducted. searching in the MEDLINE and Cochrane Oral Health Group databases for scientific articles published between January 2000 and December 2016, applying the following search terms: “alveolar bone,” “bone regeneration,” “socket preservation,” “bone preservation,” “bisphosphonates,” “pa-midronate,” “alendronate,” “zolendronic acid.” 

- Eligibility criteria 

Eligibility criteria for inclusion in the review were as follows: (a) original studies (clinical and experimental); (b) inclusion of a control group (bone remodeling without topical application of bisphosphonates); (c) intervention: effect of topical application of bisphosphonates on bone preservation/regeneration; (d) studies published in the English language. Only articles published from January 2000 to December 2016 were included. Letters to the editor, historic reviews, commentaries, case reports and in vitro studies were excluded.

- Search Strategy 

A literature search was conducted among the PubMed/Medline (National Library of Medicine, Washington, DC), EMBASE, Scopus, Web of knowledge, and Google-Scholar databases for articles published from January 2000 up to and including December 2016, using different combinations (and Boolean Operators: AND, OR, NOT) of the following search terms/key words: “topical bisphosphonates,” “bone preservation,” “bone regeneration,” “bone substitutes,” “bone graft,” “bone defects,” “bone remodeling,” “alveolar bone.” The titles and abstracts of studies identified in the search were screened by the authors (N.L.C and O.S.C.) and checked for agreement. The full texts of studies screened by title and abstract and considered to be of interest were read and evaluated independently, applying the eligibility criteria. References to any other published articles were also screened to identify potentially relevant original or review articles. Following the electronic search, a further manual search was performed in the websites of the leading scientific journals on dentistry and implant dentistry: Clinical Oral Implants Research, Clinical Oral Investigations, Clinical Implant Dentistry and Related Research, European Journal of Oral Implantology, European Journal of Prosthodontics and Restorative Dentistry, Journal of Oral Maxillofacial Surgery, Journal of Oral Surgery, Journal of Clinical Periodontology, Journal of Periodontology, Implant Dentistry, International Journal of Periodontics and Restorative Dentistry, The International Journal of Oral & Maxillofacial Implants, and European Journal of Inflammation. Again, the eligibility criteria were applied independently and any disagreement between the reviewers was resolved through discussion.

- Study Selection and Data Collection Process 

Two reviewers (N.L.C. and O.S.C) carried out the selection process, screening the articles’ titles and abstracts. The full texts of all studies of possible relevance were then obtained, and eligibility assessment and data extraction were performed independently in an unblinded standardized manner by the two authors. The data extracted included eligibility criteria, baseline characteristics, interventions, outcomes, and methodological quality. When the reviewers did not agree, a third reviewer and statistical researcher (J.L.C-G.) scored the abstracts to decide whether the article should be included or excluded. Afterwards, the full text of all the selected manuscripts were read and carefully evaluated.

- Data Items 

The information extracted from each article included: (1) type of article; (2) specimen and sample; (3) type of bisphosphonate; (4) dose of bisphosphonate; (5) scenario; (6) results. Any disagreements on data extraction were resolved by discussion between the two reviewers.

- Quality Assessment 

The methodological quality of the studies was assessed focusing on the following issues: bibliography, randomization method, examiner blinding, study population characteristics, baseline and outcome evaluations.

Two reviewers assessed the quality of each study independently. Disagreements on validity assessment were resolved by consensus and discussion; when consensus could not be reached, a third reviewer was consulted.

A study was classed as at a low risk of bias when the study population was selected randomly, when inclusion/exclusion criteria were defined, losses to follow-up reported, measurements validated, and the statistical analysis reported. If one of these five criteria was lacking, the study was classed as having a moderate potential risk of bias. If the study was lacking two or more of these criteria, it was considered as suffering a high potential risk of bias.

## Results

**Figure 1:**
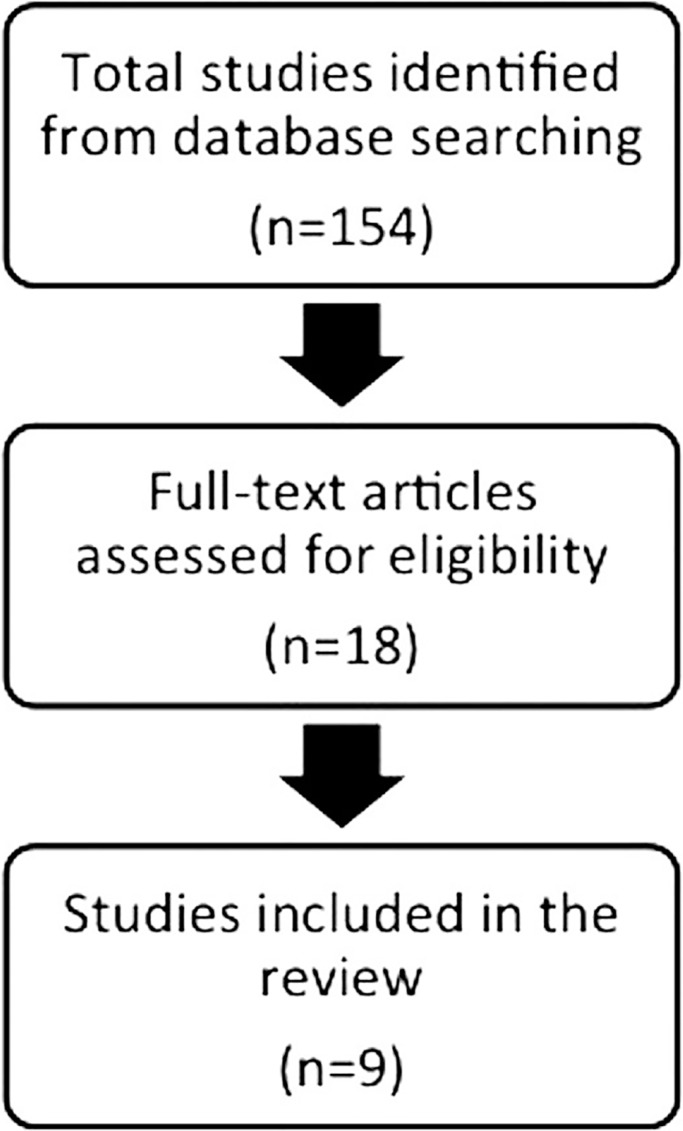
Flow chart of studies included in the review.

The initial electronic search identified 154 studies. After screening abstracts and key words, 18 potentially relevant articles were selected (agreement between reviewers 88.67%; kappa = 0.65). After reading the complete manuscripts, nine studies were excluded due to inadequate study design, absence of a control group, or because the data reported was insufficient. The manual search and cross-referencing did not locate any further articles, so the final selection included nine articles (Fig. 1).

- Selected study characteristics 

The articles that met the inclusion criteria detailed above were all animal research studies.

• Participants: the studies included involved a total of 94 rats, 8 sheep, 15 rabbits, 8 domestic pigs, and 8 Beagle dogs.

• Evaluation period: all studies had an evaluation period of at least of four weeks.

• Intervention: data from each article were analyzed and information about the study type, animals, type of bisphosphonate applied, its dose, scenario, and outcomes were extracted ( Table 1). In four out of the nine studies, the bisphosphonate used was alendronate (#1, #3, #6 and #7), in four the bisphosphonate was pamidronate disodium (#4, #5, #8 and #9), and one study applied clodronate (#2). Different bone fillers were used: allografts (#1); autografts (#2, #3); xenografts; and alloplastic materials.

**Table 1:**
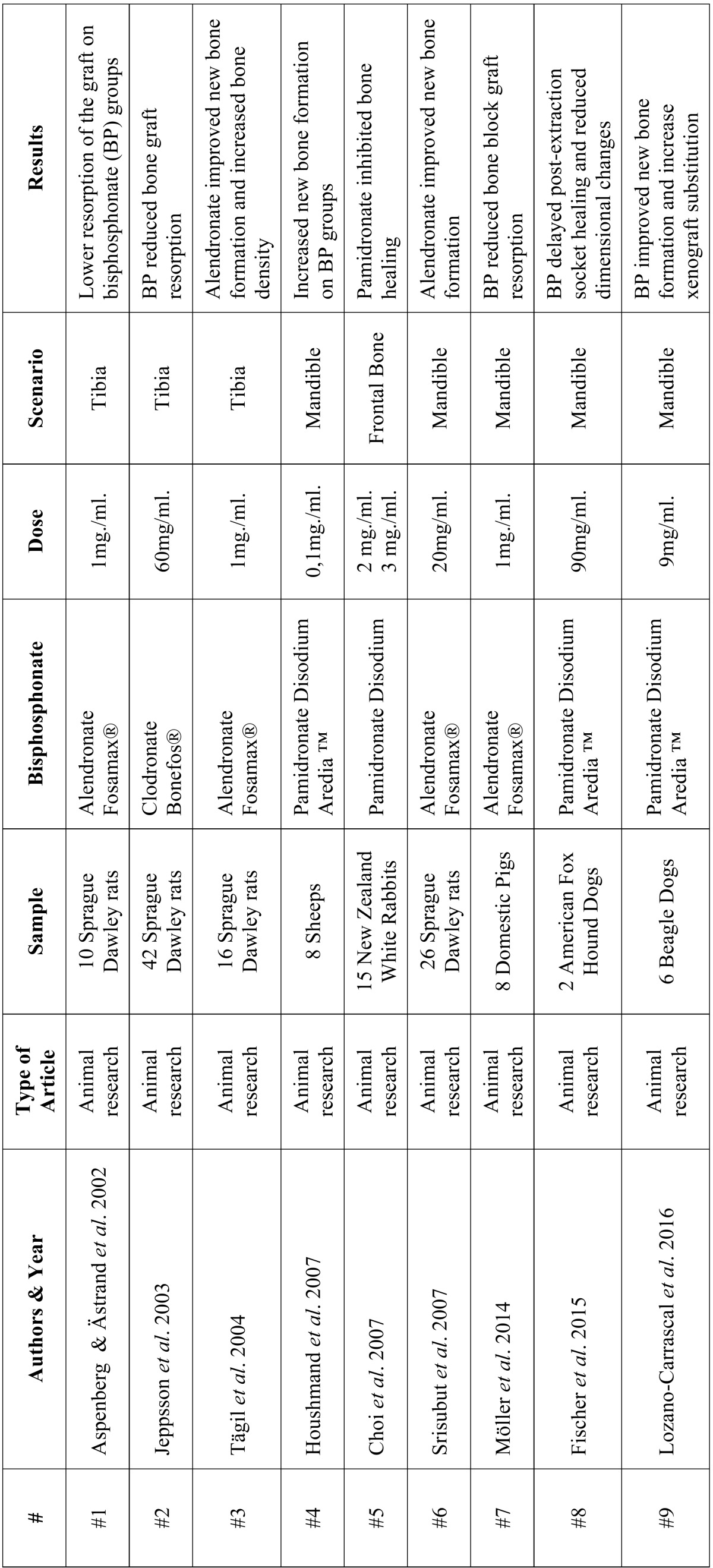
Summary of the articles finally included in this systematic review (authors, year of publication, type of article, type of bisphosphonate, dose, scenario and mean results).

• Outcomes: the outcomes reported varied greatly.

- Quality assessment 

**Figure 2:**
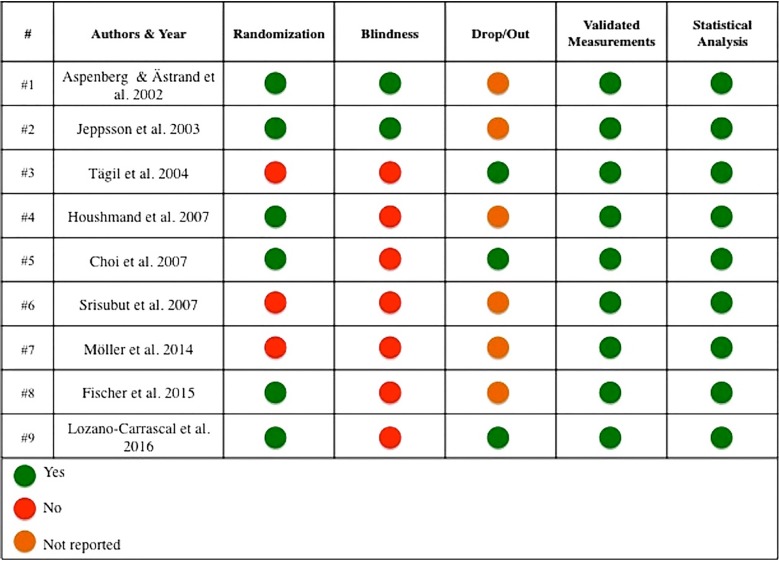
Quality and potential risk of bias assessment of the included studies.

Quality assessment of the studies analyzed is shown in Figure 2. The estimated risk of bias was considered to be moderate in four cases (#1, #2, #5, and #9) and high in five (#3, #4, #6, #7, and #8). None of the studies were considered to have a high level of evidence with an estimated low risk of bias.

- Individual study results 

It was difficult to compare the findings between studies due to the heterogeneity of study designs, the lack of consistency in the methodologies used for data collection and analysis, and the lack of concurrence between outcome definitions. Therefore, below is a more extensive overview of each article, the treatment performed, and the results obtained ( Table 1).

Aspenberg & Ästrand (25): this study evaluated the effect of the immersion of cancellous bone allografts in a bisphosphonate solution before implantation in a rat model, in a bone conduction chamber. In the experimental group, grafts were immersed in an alendronate solution (1 mg alendronate / 1 ml water) for 10 minutes, and then rinsed 3 times for 3 minutes in saline, to remove any unbound alendronate. In the control group, the grafts underwent the same treatment with saline only. In the control group chambers after 6 weeks healing, the grafts were entirely resorbed; only 22% of the space was filled by newly formed bone. In alendronate-treated specimens, grafts seemed intact, and 70% of the space was filled by graft and newly formed bone. The authors concluded that local graft treatment with a bisphosphonate appears to be risk-free, and may prevent mechanical graft failure due to resorption.

Jeppsson *et al.* (26): in this study, 10 out of 42 rats received bilateral chambers containing bone grafts from the rats’ proximal tibiae. On the experimental sides, the grafts were soaked in a clodronate solution (60 mg/ml) for 10 minutes. On the control sides, grafts were treaded with saline. After 6 weeks healing, the bisphosphonate-treated side showed increased bone density and higher graft resistance.

Tägil *et al.* (27): the authors extracted pairs of frozen cylindrical osteochondral grafts from rats’ patellar grooves; these were placed in chambers made in the proximal tibiae of 16 rats. One graft from each pair was submerged in an alendronate solution (1mg/ml) for 10 minutes. The other graft was immersed in water. After 6 weeks healing, histological examination found denser trabecular bone (42%) in alendronate-treated rats, versus 20% in untreated control samples. The authors concluded that the topical application of alendronate reduced the risk of collapse of osteochondral grafts, during revascularization and bone remodeling.

Houshmand *et al.* (28): this study evaluated the capability of pamidronate disodium to enhance bone regeneration of bovine-derived hydroxyapatite placed in infrabony defects in eight sheep. Three defects were prepared: (negative-control group) unfilled; (positive-control group) filled with bovine-derived hydroxyapatite (Bio-Oss®) alone; (case group) bovine-derived hydroxyapatite (Bio-Oss®) mixed with pamidronate disodium (1 mg of pamidronate disodium was dissolved in 10 ml of sterile distilled water and mixed with 1 gr of bovine-derived hydroxyapatite). After 6 weeks healing, the cavities of the case group showed significantly higher amounts of bone formation, and fewer osteoclasts and xenograft particles embedded in the regenerated bone. The authors concluded that adding pamidronate disodium to a demineralized bovine-derived hydroxyapatite improved the osteoconductive and regenerative capacity of the biomaterial.

Choi *et al.* (29): these authors mixed a high-dose topical application of pamidronate with L-lactide-co-glycolide (PLGA) as carrier material. The study included 15 rabbit calvaria bone defects. Four defect groups were created in each rabbit calvaria: (1) untreated bone defect; (2) PLGA only; (3) 2 mg of pamidronate with PLGA; and (4) 3 mg of pamidronate with PLGA. In radiographic analysis, radiopacity was lower in pamidronate groups at 1, 2, 4, 6 and 8 weeks after surgery. In histological analysis, after 2-8 weeks healing, the amount of newly formed bone was lower in pamidronate groups, and signs of avascular necrosis were observed. The authors concluded that pamidronate inhibited bone healing, which the authors explained was due to the blocking of angiogenesis, and/or inhibition of osteoclast activity, necessary for bone healing.

Srisubut *et al.* (30): created 5 mm diameter bone defects in the mandible angle of 26 rats. In the experimental group, bioactive glass was mixed with an alendronate solution (20 mg alendronate / 1 ml saline) and placed in the defects; in the control group, the bioactive glass was soaked with physiological saline. Four weeks after surgery, no statistically significant differences were found in the number of osteoclasts or the lesion sizes between the two groups. The experimental group showed a significantly higher amount and percentage of new bone formation.

Moller *et al.* (31): experimented with topical applications of alendronate aqueous solution (1mg/ml) to prevent the surface resorption of onlay bone grafts in eight adult pigs: (1) in combination with a collagen membrane (Bio-Gide®); (2) mixed with bovine bone mineral (Bio-Oss®); (3) or applied directly to autologous bone grafts. The same materials without bisphosphonates were used as controls on the contralateral side. After 3 months healing, significantly lower loss of graft height was seen on the test side for Bio-Gide® + alendronate, Bio-Oss® + alendronate, and bone graft + alendronate versus Bio-Gide®, Bio-Oss® and bone graft alone, respectively. In five cases, necrosis of the overlaying periosteal tissues with alendronate was observed macroscopically. The authors concluded that bisphosphonate-treated membrane or bovine bone mineral reduced bone graft resorption; however, the risk of periosteal necrosis demands better adaptation of the dose.

Fischer *et al.* (32): placed collagenated porcine bone substitute (Osteobiol Gen-Oss; CPB) rehydrated with 90 mg/ml pamidronate (test), or with sterile saline (control) in post-extraction sockets in two American foxhound dogs. After 4 months healing, they observed limited amounts of bone at test sites. The combination appeared to delay extraction socket healing and to obstruct the resorption of the porcine bone substitute. In contrast, it seemed to reduce post-extraction dimensional changes in terms of hori-zontal bone width, which was nearly three times higher at control sites, compared with sites treated with pamidronate.

Lozano-Carrascal *et al.* (33): this study used six Beagle dogs. Small (SD) and large defects (LD) were created in both quadrants of the lower jaw. Using a randomized design, the alveoli corresponding to the right hemi-mandible were used as controls (C) and were filled with MP3® porcine collagenated bone (OsteoBiol™) after rehydration with sterile saline. The left hemi-mandible defects were filled with MP3® prehydrated with pamidronate solution (9 mg/ml). After 4 and 8 weeks healing, histomorphometric analysis revealed greater new bone formation and lower residual graft particles for both SD and LD test groups, compared with SD and LD control groups, respectively. The authors concluded that porcine xenografts modified with pamindronate favor new bone formation and increased porcine xenograft substitution/replacement.

## Discussion

The biological effects of bisphosphonates are many and varied. Recent data drawn from *in vivo* and *in vitro* studies have demonstrated that they act not only by inhibiting bone resorption mediated by osteoclasts but also have the capacity to stimulate osteoblast differentiation and activity, and therefore to enhance new bone formation (12,13). But these properties depend on the means of administration, concentration, and the active principle used (4).

Topical application of an amino-bisphosphonate solution on bone defects or post-extraction sockets, whether alone or mixed with a bone graft, appears to be a risk-free procedure, according to most of the articles analyzed in the present review. With this means of administration, the bisphosphonates act on the early phases of bone healing and are mainly absorbed by the adjacent bone, so that only a small part of the total amount is released into circulation.

The main disadvantage of bone autografts or allografts is the unpredictability of resorption (34). But topical pre-treatment of a graft with a bisphosphonate solution can prevent mechanical graft failure caused by resorption (31). Moreover, once the graft surface has been covered by newly formed bone, this seems to protect against bone resorption, increasing new bone formation and bone density (25-27).

Bisphosphonates also improve the regenerative capacity of biomaterials. Some authors (28,31,33) observed improved osteoconductive properties of bovine or porcine-derived xenografts when mixed with low doses of bisphosphonates, as histomorphometric analysis revealed significantly higher amounts of new bone formation and less xenograft particles surrounded by the regenerated bone.

Although most of the studies reviewed confirmed the positive effects of bisphosphonates on new bone formation, even at high doses (30), others observed delayed bone healing and lower amounts of newly formed bone, with some signs of avascular necrosis (29,32). This discrepancy between results might be explained by methodological differences, especially in terms of the active principle, dosage, and follow-up duration.

Bisphosphonates have been shown to reduce post-extraction dimensional changes (32), to increase new bone formation (27,30,33), and to boost the action of biomaterials, stimulating bone regeneration (25,26,28,31). These outcomes have great clinical relevance in situations in which it is necessary to enhance new bone formation. But in spite of these positive observations, they should be treated with caution given the heterogeneity of the studies, deriving from wide variations in methodology, surgical procedure, and/or healing periods.

Figure 2 shows that the estimated risk of bias was considered to be moderate in four studies (#1, #2, #5 and #9) and high in five (#3, #4, #6, #7 and #8). None of the studies were considered to present the highest level of evidence and so a low estimated risk of bias. Although all the studies were performed with validated measurement and statistical analysis, only six articles were randomized. Two out of the six (#1 and #2) were randomized and blind, but failed to report any dropouts. Only one article (#9) ex-plained the randomization method. Three studies (#3, #6 and #7) were not randomized. Only two studies (#4 and #5) were carried out with positive and negative control groups, the rest were performed with test and control groups. All the articles explained the type and dose of bisphosphonate used, but only one article (Houshmand *et al.*) (28) (#5) reported the amount of bone graft material mixed with bisphosphonate solution in detail.

No human studies were found in the literature search and there is a lack of information regarding the long-term longevity of regenerated defects. From the results obtained, it is impossible to determine which type of defect, surgical technique, type of bisphosphonate, dose, bone graft, or healing period provides positive outcomes in the long-term. Furthermore, there is little data regarding the possible influence of these treatments on the success/survival rates of implant therapies. In this context, it would be unwise to recommend any particular technique until more research has been published. Future studies must offer well-designed trials that are randomized and blinded, reproducible, with validated evaluation methods, and complete details of the materials and methods used.

## Conclusions

In spite of the heterogeneity of methodologies and the high risk of bias among the animal research studies included in the present review, the topical application of bisphosphonate solution would appear to:

- Reduce alveolar bone resorption and increase new bone formation in alveolar bone defects.

- Boost the regenerative capacities of biomaterials, favoring particle substitution, and increasing bone density.
